# Automatic detection of patient-ventilator asynchrony by spectral analysis of airway flow

**DOI:** 10.1186/cc10309

**Published:** 2011-07-12

**Authors:** Guillermo Gutierrez, Guillermo J Ballarino, Hulya Turkan, Juan Abril, Lucy De La Cruz, Connor Edsall, Binu George, Susan Gutierrez, Vinayak Jha, Jalil Ahari

**Affiliations:** 1 Pulmonary and Critical Care Medicine Division, The George Washington University MFA, 2150 Pennsylvania Ave, NW, Washington, DC 20037, USA

## Abstract

**Introduction:**

Adequate ventilatory support of critically ill patients depends on prompt recognition of ventilator asynchrony, as asynchrony is associated with worse outcomes.

We compared an automatic method of patient-ventilator asynchrony monitoring, based on airway flow frequency analysis, to the asynchrony index (AI) determined visually from airway tracings.

**Methods:**

This was a prospective, sequential observational study of 110 mechanically ventilated adults. All eligible ventilated patients were enrolled. No clinical interventions were performed. Airway flow and pressure signals were sampled digitally for two hours. The frequency spectrum of the airway flow signal, processed to include only its expiratory phase, was calculated with the Cooley-Tukey Fast Fourier Transform method at 2.5 minute intervals. The amplitude ratio of the first harmonic peak (H_1_) to that of zero frequency (DC), or H_1_/DC, was taken as a measure of spectral organization. AI values were obtained at 30-minute intervals and compared to corresponding measures of H_1_/DC.

**Results:**

The frequency spectrum of synchronized patients was characterized by sharply defined peaks spaced at multiples of mean respiratory rate. The spectrum of asynchronous patients was less organized, showing lower and wider H_1 _peaks and disappearance of higher frequency harmonics. H_1_/DC was inversely related to AI (*n *= 110; r^2 ^= 0.57; *P *< 0.0001). Asynchrony, defined by AI > 10%, was associated H_1_/DC < 43% with 83% sensitivity and specificity.

**Conclusions:**

Spectral analysis of airway flow provides an automatic, non-invasive assessment of ventilator asynchrony at fixed short intervals. This method can be adapted to ventilator systems as a clinical monitor of asynchrony.

## Introduction

Patient-ventilator asynchrony occurs frequently in mechanically ventilated patients, in particular those with acute or severe lung injury [1,2]. Asynchronous events occur when a patient's intrinsic respiratory rhythm fails to entrain to machine inflation or when ventilatory support is inadequate to meet the patient's requirements. Poorly synchronized patients remain on mechanical ventilation longer [3] and have worse outcomes [4].

The most reliable method presently available to detect asynchrony is the placement of a balloon catheter in the esophagus to measure intra-thoracic pressure changes during the breath cycle [5]. Electromyography also has been used to assess asynchrony by comparing ventilatory muscle electrical activity to the initiation of ventilator-delivered inspiratory flow [6]. Both methods have the disadvantage of being invasive and not well tolerated by some patients, particularly those who are alert. Non-invasive methods to establish the degree of patient-ventilator synchrony have been proposed as possible alternatives to electromyography and measures of intrathoracic pressures. Perhaps the method with the widest clinical acceptance is the computation of an asynchrony index (AI) by visual analysis of airway flow and pressure tracings [3]. Although useful as a research tool, the calculation of an AI is time consuming and does not lend itself to real-time monitoring of asynchrony in mechanically ventilated patients.

Airway flow and pressure are periodic functions whose frequency spectra can be determined using Fourier transformation. This method separates a time dependent signal into an infinite number of sine and cosine waves whose frequencies and amplitudes are displayed as a frequency spectrum. We hypothesize that application of spectral frequency analysis to airway signals will allow for the detection of patient-ventilator asynchrony in a non-invasive and automatic manner.

**Figure 1:**
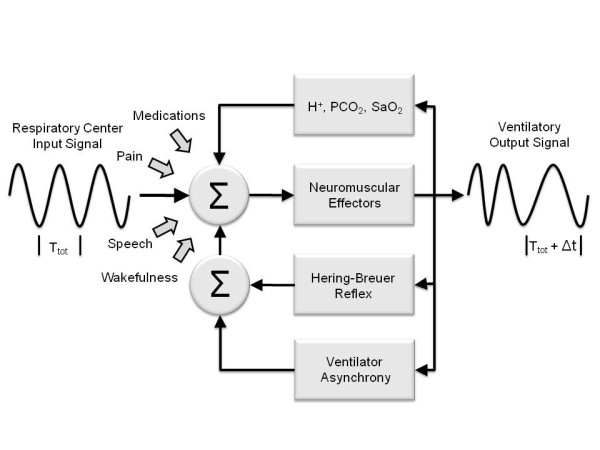
**A conceptual model of the feedback loops producing breath-by-breath changes in respiratory periodicity**. See text for explanation. Δt, breath-by-breath timing variations; S_a_O_2_, arterial O_2 _saturation; T_Tot_, total breath cycle time.

The physiological notion underpinning the use of frequency spectral analysis as a measure of patient-ventilator asynchrony is shown in Figure 1. Although the precise cellular and molecular mechanisms that regulate the periodicity of respiratory rhythm are largely unknown, the respiratory center is thought to generate a pacemaker signal with nearly constant T_Tot _[7]. This pacemaker signal is modulated by cortical inputs, such as the degree of alertness, speech, pain, and so on, and also by changes in tidal volume and respiratory rate that activate chemical and mechanical feedback loops. The various inputs produce breath-by-breath timing variations in T_Tot _denoted in the diagram by the term Δt. Under normal conditions Δt is small, imparting the respiratory cycle with its inherent timing variability [8,9].

Mechanical ventilation appears to impose an additional feedback loop on breathing pattern, possibly acting through the fast-acting Hering-Breuer mechanoreceptor reflex [10]. Asynchronous events, whether occurring during inspiration or expiration, are likely to increase mechanoreceptor firing, resulting in increased breath-by-breath T_Tot _variability [11]. Whereas subtle variations in T_Tot _are difficult to detect from direct examination of airway signal tracings, these changes can be readily identified from the signal's frequency spectrum.

To test the hypothesis that time series analysis of airway flow provides a noninvasive assessment of patient-ventilator asynchrony, we sampled airway signals digitally in mechanically ventilated patients during a two-hour observation period and compared the frequency spectra of airway flow to corresponding AI values.

## Materials and methods

This was a prospective, observational study conducted at The George Washington University Hospital intensive care unit from February 2010 to January 2011. We chose an arbitrary sample of 110 patients of either sex, 18 years of age or older, who were mechanically ventilated on a Servo*i *or Servo*s *Maquet ventilator (Maquet Critical Care AB, Solna, Sweden). The study was approved by the GWU Institutional Review Board (IRB#110910) and informed consent to participate was obtained from the patient or surrogate. All those mechanically ventilated of whom informed consent was granted were enrolled in the study. All patients were monitored hemodynamically. This was strictly an observational study and all treatment modalities, including ventilatory mode and ventilator settings, were determined by treating physicians not involved in the study.

### Data acquisition

Data were acquired during the two-hour period that followed enrollment in the study. We measured airway flow and pressure continuously, using the built-in data acquisition system of the Servo ventilator (Servo*i*/Servo*s *Computer Interface Emulator, Sölna, Sweden) programmed to sample airway signals digitally at 30 Hz (equivalent to 1,800 cycles per minute), a sampling rate exceeding the Nyquist criterion for respiratory signals [12]. We also sampled O_2 _saturation (S_p_O_2_) by pulse oximetry, arterial blood pressure from an arterial line, and heart rate from one electrocardiographic lead. These signals were acquired from the analog output port of the ICU monitor (Tram^® ^Multi-Parameter Module, GE Healthcare Bio-Sciences Corp., Piscataway, NJ, USA) with an analog-to-digital converter at 30 Hz (DI148U A/D, DATAQ Instruments, Inc. Akron, OH, USA).

### Data analysis

The sampled airway flow signal was modified by setting all inspiratory (positive) values to zero. This resulted in a periodic, continuous signal displaying only the expiratory phase of the breathing cycle. The Discrete Fourier Transform of the processed flow signal was obtained with the Cooley-Tukey Fast Fourier Transform (FFT) algorithm [13]. Since this method requires input data in blocks of 2^n ^samples, we applied the FFT to segments containing 4,096 consecutive samples. The data segments encompassed approximately 2.3 minutes of observation and produced one distinct frequency spectrum with a frequency resolution of 7.32 × 10^-3 ^Hz. To avoid the possibility of spectral leakage, our program insured that each sampling window contained an integer number of cycles beginning at the initiation of inspiration. Spectra were generated at 2.5 minute intervals, for a total of 48 spectra during the two-hour observation period. The amplitude of the first harmonic peak (H_1_) was calculated with a peak detection algorithm based on Lorentzian peak analysis [14]. We calculated the amplitude ratio of H_1 _to that of zero frequency or DC component (H_1_/DC ratio) for each spectrum. Hemodynamic and ventilatory variables were averaged every 2.5 minutes and monitored for 2 hours. Data acquisition and frequency spectral analysis were performed in real time at bedside using a laptop computer with software written in-house specifically for this purpose (Visual Basic Programming Language, Microsoft Corporation, Redmond, WA, USA).

The AI values were computed visually [3,4] from the flow and pressure recordings corresponding to the time window used to produce one frequency spectrum. Three trained observers, who were blinded to the results of the spectral analysis, calculated AI at times 0, 30, 60, 90 and 120 minutes. The mean of the three AI measurements was taken as the AI value at each time point. For each patient we obtained the time-averaged AI value for times 0, 30, 60, 90 and 120 minutes and plotted them as functions of their corresponding time-averaged H_1_/DC values. We conducted a Cohen Kappa test modified by Fleiss for three independent observers [15] and found a κ statistic = 0.66 when comparing the ability of the three observers to detect asynchrony index values > 10% [3]. According to Landis and Koch [16], this κ value corresponds to substantial agreement among the three observers.

### Statistics

We tested for significant differences between distributions of independent samples with the Mann-Whitney *U *test for nonparametric data. The Chi Square test with Pearson's correction was used to test for differences in categorical variables. A receiver operating characteristic curve (ROC) was constructed for H_1_/DC, in which asynchrony was defined by simultaneously measured AI values > 10% [17]. The relationship between dependent variables was determined with linear regression [18]. Numerical data are shown as median and interquartile range and *P *< 0.05 was considered significant.

## Results

Patient demographics, most common ICU admission diagnoses, and conditions leading to the initiation of mechanical ventilation of the 110 mechanically ventilated patients enrolled in the study are shown in Table 1. There was a preponderance of male patients. Patients were enrolled early upon their admission to the ICU and initiation of mechanical ventilation. These were acutely ill patients with a high Simplified Acute Physiology Score (SAPS) II score of 57, carrying a predicted mortality rate of 62%. There was an almost equal distribution between medical and post-operative patients with sepsis being the most common major diagnosis. Acute lung injury was the condition most frequently resulting in mechanical ventilation. Patients were ventilated using the following modes: assisted pressure controlled (PC; *n *= 18); pressure regulated volume controlled (PRVC; *n *= 55); assisted volume controlled (VC; *n *= 26); and pressure support ventilation (PS; *n *= 11).

**Table 1:** Patient demographics, major diagnoses, and primary causes for mechanical ventilation (*n *= 110)

Age (years)	60 (51 to 72)
Male gender	66.4%
Days in Hospital	4.0 (2.0 to 9.8)
Days in ICU	3.0 (1.0 to 7.0)
Days on Mechanical Ventilation	2.0 (1.0 to 5.8)
Enrollment SAPS II	57 (52 to 68)
Enrollment SOFA	6 (5 to 9)
**Major diagnoses:**	
Cardiogenic shock	11.8%
COPD	7.3%
Post operative	43.6%
Sepsis	34.5%
**Conditions resulting in mechanical ventilation:**	
Acute lung injury	37.3%
Airway protection	12.7%
Pneumonia	12.7%
Post-op complication	10.9%
Pulmonary edema	14.5%
Respiratory failure*	10.0%

*Defined as severe hypercarbia or hypoxemia in the absence of airspace densities in the chest roentgenogram. Numerical data are shown as median and interquartile range. SAPS, Simplified Acute Physiology Score; SOFA, Sequential Organ Failure Assessment.

**Figure 2:**
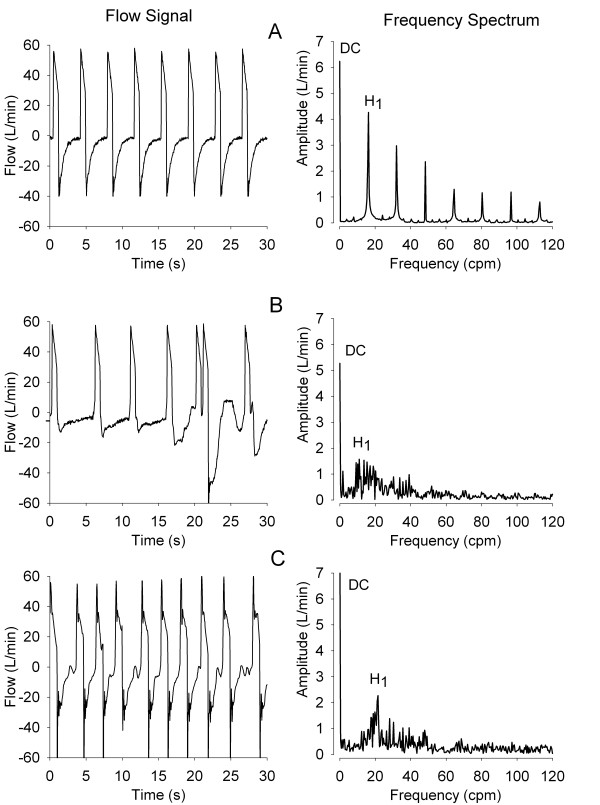
**Airway flow signals and their corresponding frequency spectra**. Frequencies shown as cycles per minute (cpm). H_1 _denotes the first harmonic peak amplitude and DC is the amplitude of the zero frequency component. Panel **A **illustrates a condition of patient-ventilator synchrony in which the spectral pattern is characterized by sharp peaks located at multiples of the respiratory rate. Panel **B **shows double triggering asynchrony during inspiration. Compared to that of Patient A, the frequency spectrum of Patient B has lost much of its initial organization. Panel **C **displays ineffective triggering asynchrony during expiration. The corresponding frequency spectrum also shows loss of organization and diminished H_1 _amplitude; cpm, cycles per minute; DC, zero frequency amplitude; H_1_, first harmonic peak amplitude; L/minute, liters per minute; s, seconds.

Figure 2 shows airway flow signals and corresponding frequency spectra during conditions of synchrony and asynchrony. Panel A shows a synchronous flow signal. The frequency spectrum of this signal is characterized by a finite zero frequency component (DC) with an amplitude equal to mean expiratory flow. This is followed by a series of sharp, Lorentzian-shaped peaks displaying progressively lower amplitude. The first harmonic peak, denoted as H_1_, is located at a frequency equal to the mean respiratory rate of the data segment used to develop the frequency spectrum. Subsequent peaks are located at frequency multiples of the mean respiratory rate. The H_1_/DC amplitude ratio corresponding to this particular spectrum is 67.4%. Panel B shows a patient experiencing asynchrony characterized by double triggering during inspiration. Compared to that of Patient A, the frequency spectrum of Patient B shows loss of organization, with greatly diminished H_1 _amplitude and the virtual absence of subsequent harmonic peaks. The H_1_/DC for this case is 29.7%. Panel C shows asynchrony manifested by ineffective triggering during expiration. The frequency spectrum for this condition is similar to that for Panel B, with H_1_/DC = 24.8%.

**Figure 3:**
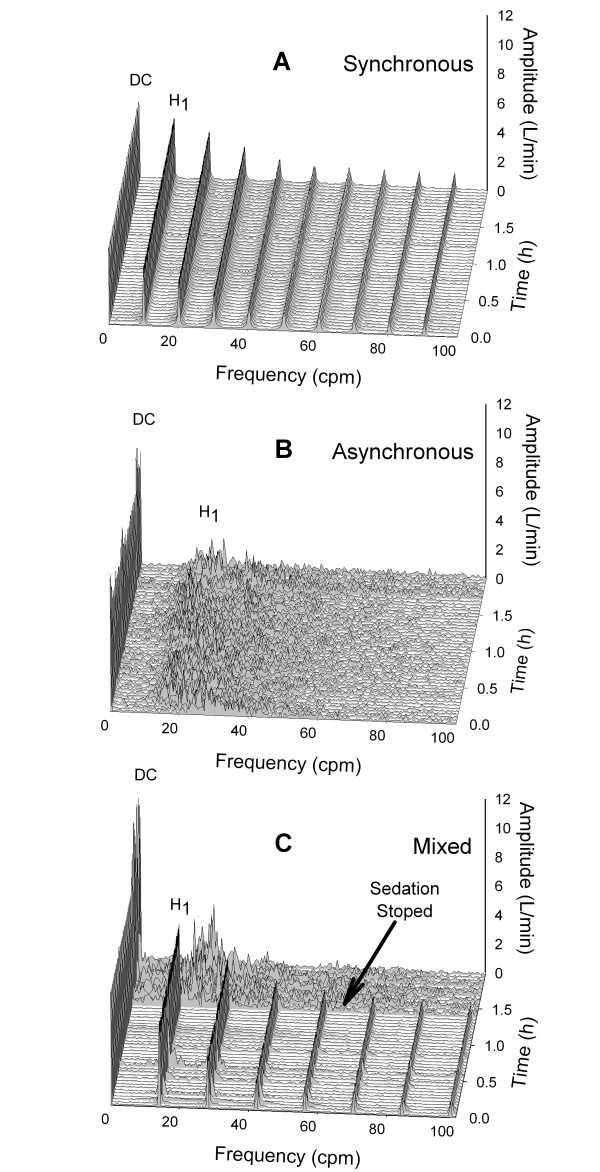
**Waterfall ensembles featuring 48 spectra during the two-hour period of observation**. Frequencies shown as cycles per minute (cpm). Panel **A **corresponds to a patient with no asynchrony. Panel **B **is that of an asynchronous patient. Panel **C **is an initially synchronous patient who develops asynchrony upon cessation of sedation. cpm, cycles per minute; DC, zero frequency amplitude; h, hours; H_1_, first harmonic peak amplitude; L/minute, liters per minute.

Frequency spectra during the two-hour observation period varied according to the degree of patient-ventilator asynchrony. This is illustrated in Figure 3, where staggered spectral ensembles are shown at 2.5-minute intervals for the 2-hour observation period. Patient A displayed a synchronous pattern that replicated almost exactly during the two-hour period. The average AI and H_1_/DC values during that time were 0% and 79.5%, respectively. Conversely, Patient B appeared to be asynchronous during the whole two-hour observation period, with a mean AI of 29.5%. The corresponding frequency spectra displayed a persistently disorganized pattern with mean H_1_/DC of 23.6%. Patient C displayed a mixed pattern. During the first 1.4 hours of observation Patient C appeared to be synchronous with the ventilator, with mean AI = 2.0. Corresponding frequency spectra appeared organized with H_1_/DC = 69.5%. Following the cessation of sedation, and for the remainder of the observation period, the patient's AI increased to a mean of 38.2%, suggesting the development of significant asynchrony. The corresponding frequency spectra became less organized, evolving into an asynchronous pattern with increases in DC and a shift of H_1 _to a higher frequency, signifying increased mean expiratory flow and faster respiratory rate, respectively. Mean H_1_/DC during that time was 23.4%.

**Figure 4:**
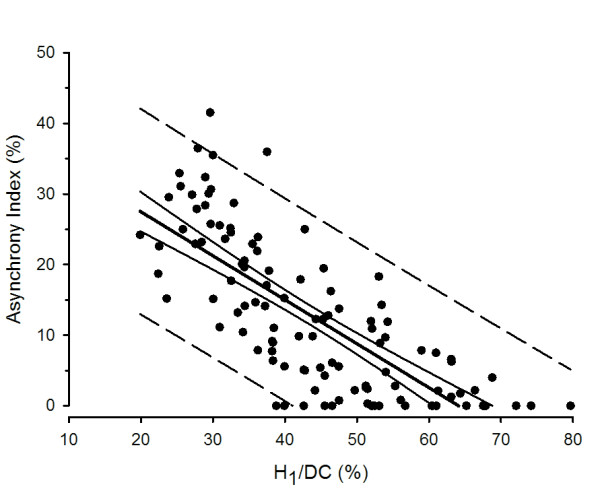
**Asynchrony Index (AI) as a function of H_1_/DC**. The average synchrony index (AI) of measurements taken at 0, 30, 60, 90 and 120 minutes plotted as a function of corresponding average H_1_/DC for each study patient (*n *= 110). H_1_/DC varied inversely with AI during the two-hour during observation period (AI, 39.9 - 0.6 H_1_/DC; r^2 ^= 0.57, *P *< 0.0001). DC, zero frequency amplitude; H_1_, first harmonic peak amplitude.

Figure 4 shows the mean AI and H_1_/DC values during the two-hour observation period for each study patient (*n *= 110). Each point represents the average of five AI and H_1_/DC determinations obtained at 0, 30, 60, 90 and 120 minutes. Also shown are the 95% confidence bands for the regression equation and the prediction interval encompassing 95% of the data. There is an inverse relationship between AI and H_1_/DC (AI = 39.9 - 0.6 H_1_/DC; r^2 ^= 0.57, *P *< 0.0001), a finding that supports the stated hypothesis that decreases in H_1_/DC are associated with patient-ventilator asynchrony, as determined by AI.

**Figure 5:**
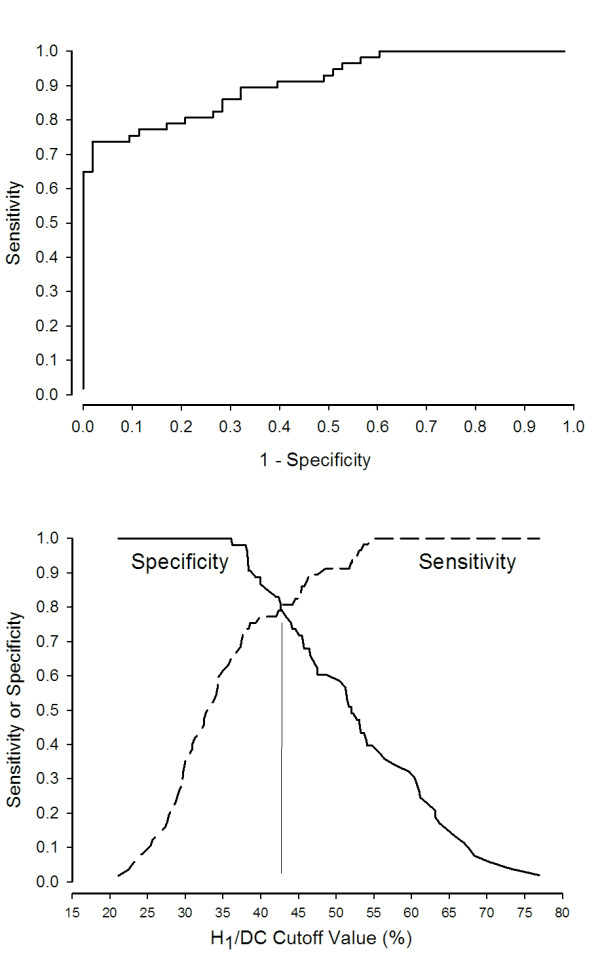
**Receiver Operating Characteristic (ROC) and sensitivity analysis**. (Top) ROC curve for H_1_/DC. Area under the curve is 0.91 ± 0.03 (*P *< 0.0001). (Bottom) Sensitivity and specificity plotted as functions of H_1_/DC values. The curves intersect at H_1_/DC = 42.9%, a value that identifies asynchrony (as defined by AI > 10%) with sensitivity and specificity of 82.7% each. DC, zero frequency amplitude; H_1_, first harmonic peak amplitude.

The ROC curve for H_1_/DC, using AI > 10% as an indicator of asynchrony, is shown in Figure 5 (Top). The area under the ROC is 0.91 ± 0.03 (*P *< 0.0001). The bottom graph plots sensitivity and specificity as functions of H_1_/DC cutoff values. The curves intersect at H_1_/DC = 42.9%, a value that identifies asynchrony (as defined by AI > 10%) with sensitivity and specificity of 82.7% each.

There were no significant differences in the overall prevalence of asynchrony as determined by AI (50.9%) or H_1/_DC (51.8%). We also found no differences in the prevalence of asynchrony when patients were classified according to mode of ventilation (Table 2). Patients ventilated with PS experienced significantly more asynchrony when compared to other ventilatory modes (*P *< 0.001). The cutoff value for H_1/_DC of 43% identified as asynchronous 47 of the 56 individuals having AI > 10% (84% sensitivity). In those 47 individuals, the most common types of asynchronies displayed during the two-hour period of observation were ineffective triggering during inspiration (*n *= 17; 36.2%); ineffective triggering during expiration (*n *= 16; 34.0%); and double triggering (*n *= 14; 29.8%).

**Table 2:** Prevalence of total asynchronies and types of asynchrony according ventilatory mode

		Asynchrony Prevalence (%)	
**Mode**	**n**	**H_1_/DC**	**AI**

PC	17	50.0	55.6
PRVC	56	49.1	50.9
VC	26	42.3	26.9
PS	11	90.9 †	100.0 †

IT, ineffective triggering; Mode, mode of mechanical ventilation; n, number of patients ventilated with a given mode; PC, pressure controlled; PRVC, pressure regulated volume control; PS, pressure support; VC, volume controlled; † *P *< 0.001 when compared to other modes of ventilation.

Table 3 shows ventilatory and hemodynamic variables measured during the two-hour observation period, listed according to the cutoff value for H_1_/DC of 43%. Other than a trend towards greater use of continuous i.v. sedation in the group identified as synchronous by H_1_/DC ≥43% (*P *= 0.08), there were no significant differences in any of the variables measured. We also found no differences in the distribution of major diagnoses or primary reasons for initiation of mechanical ventilation when asynchrony was detected with H_1/_DC < 43% or AI > 10% (data not shown).

**Table 3:** Ventilatory and hemodynamic variables during the two-hour observation period

	H_1_/DC < 43%	H_1_/DC ≥43%
Number of patients	57	53
Enrollment SAPS II	56.0 (53.0 to 66.0)	57.5 (50.0 to 70.0)
Enrollment SOFA	6 (4 to 8)	7 (5 to 9)
H_1_/DC (%)	34.1 (28.9 to 38.2)	53.1 (47.5 to 61.0) †
AI (%)	20.0 (11.1 to 25.5)	2.8 (0 to 8.9) †
F_I_O_2 _(%)	40.0 (39.8 to 50.0)	49.8 (40 to 57.8)
SpO_2 _(%)	97.9 (95.8 to 99.0)	97.4 (95.6 to 98.9)
RR (bpm)	18.7 (16.0 to 22.0)	17.5 (14.0 to 22.6)
MIP (cmH_2_O)	10.4 (8.8 to 12.9)	10.3 (8.4 to 14.7)
PeakP (cmH_2_O)	25.2 (20.5 to 32.1)	27.4 (22.5 to 31.3)
V_T _(mL)	502 (450 to 560)	470 (410 to 546)
PEEP (cmH_2_O)	5.0 (4.7 to 5.5)	5.1 (4.9 to 5.4)
HR (bpm)	89.8 (78.8 to 98.3)	86.6 (77.0 to 97.0)
MAP (mmHg)	80.3 (71.9 to 97.0)	78.7 (70.7 to 95.7)
Vasoactive agents (%)	29.8	43.4
Continuous sedation (%)	59.6	75.5

AI, Asynchrony Index (%); F_I_O_2_, inspired O_2 _concentration; H_1_/DC, ratio of first harmonic peak amplitude to DC component amplitude (%); HR, heart rate; MAP, mean arterial pressure; MIP, mean inspiratory pressure; PeakP, peak inspiratory pressure; PEEP, positive end expiratory pressure; RR, respiratory rate; SAPS, Simplified Acute Physiology Score; SOFA, Sequential Organ Failure Assessment; SpO_2_, arterial O_2 _saturation by pulse oximetry; V_T_, tidal volume. Data shown as median and IQR; † *P *< 0.01.

## Discussion

The purpose of the present study was to compare a novel method of asynchrony detection, based on frequency spectral analysis of airway flow, to AI, an accepted parameter of asynchrony. We studied a heterogeneous group of 110 mechanically ventilated patients and found a significant inverse correlation between the methods. Moreover, spectral analysis of airway flow could detect asynchronies with a high degree of sensitivity and specificity. Defining asynchrony by AI > 10%, a cutoff value of H_1_/DC = 43% identified asynchrony with 83% sensitivity and specificity, each.

Synchronous patients displayed a spectral pattern characterized by a series of Lorentzian shaped peaks [14] monotonically spaced at frequency multiples of the respiratory rate. Conversely, patient-ventilator asynchrony was associated with a less organized spectral pattern in which H_1 _bandwidth widened, its amplitude decreased, and higher frequency harmonics disappeared.

The zero frequency amplitude, or DC component, is defined as the time average of a periodic signal. Since the time average of air flow during the breath cycle (inspiratory and expiratory phases) is zero, its spectrum lacks a DC component. Modifying the flow signal to contain only the expiratory phase resulted in a frequency spectrum with a finite DC component. This DC component can be used to gauge sequential changes in H_1 _as the denominator for the parameter H_1_/DC. This parameter has a solid physiological foundation since the DC component equals mean expiratory flow and the frequency of H_1 _is the mean respiratory rate. In addition, the shape of the expiratory flow signal is independent of the manner used to insufflate the lungs; therefore, its frequency spectrum should not be affected by the mode chosen to ventilate the patient. This notion is supported by the results of Table 2 showing that the ability of H_1_/DC to detect asynchrony was unaffected by the mode of ventilation.

Asynchrony is associated with longer duration of mechanical ventilation and worse outcome [4]. The major types of asynchrony include double triggering, present when the inspiratory efforts of patient and ventilator are out of phase; ineffective triggering during inspiration, when the patient's flow demands are not met by the ventilator; and ineffective triggering during expiration [19]. We focused on the expiratory portion of the flow signal, but it should be stressed that the method presented here responds to asynchronies occurring both during inspiration and expiration. Spectral analysis detects small breath-to-breath variations in T_Tot _resulting from stretch receptor activation at any time during the ventilatory cycle. As far as the frequency analysis method is concerned, it does not matter whether the asynchronous event occurs during inspiration or expiration, or what type of asynchrony is encountered: trigger, flow or expiratory asynchrony. What matters is the effect that these asynchronies have on breath-by-breath T_Tot _variability. This was amply demonstrated by the similar prevalence rates for all types of asynchrony detected both by spectral analysis and by AI, whether they occurred during the inspiratory or expiratory portion of the breathing cycle.

There is no universal agreement on the prevalence of ventilator asynchrony. Chao *et al*. [4] reported a prevalence of 10%, Thille *et al*. 24% [3], and Colombo *et al*. 36% [20]. We found the asynchrony prevalence in our patient population to be approximately 50%. It is possible that this high asynchrony prevalence is related to the high illness acuity of our patient population and is in line with those reported by Piquilloud *et al*. [21] of 63.5% in patients on PS ventilation studied three days post-intubation. In particular, we noted a high prevalence of asynchrony in patients ventilated with PS. This finding is difficult to generalize, given the few individuals on PS in the study (*n *= 11). It is possible that PS ventilation may be associated with a greater degree of asynchrony than other modes of ventilation [21], but also it may be that PS ventilation was applied improperly in this cohort, a situation beyond our control given our role as observers. The relevant issue regarding our study is that detection of asynchrony in PS patients by AI (11/11) was similar to that of H_1_/DC (10/11).

Several automatic, noninvasive methods have been developed to detect patient-ventilator asynchrony. These methods rely on the analysis of airway signals for anomalies indicative of ineffective patient triggering (IT). Mulqueeny *et al*. [22] proposed applying a noise filter and an unintentional leak compensation algorithm to the flow and pressure curves, followed by the calculation of the first and second derivatives of the flow signal. They tested their method in 20 mechanically ventilated patients and found 91% sensitivity and 97% specificity when compared to the manually derived AI. Cuvelier *et al*. [23] developed an algorithm that analyzed phase portraits, a geometrical depiction of temporal changes in patient-ventilator interaction. They were able to identify 95% of all IT efforts when comparing the results of this method to esophageal tracings in 14 children with cystic fibrosis on non-invasive ventilation.

Chen *et al*. [24] developed a computerized algorithm based on small deflections of the flow and pressure signals during the expiratory phase of ventilation. The algorithm detected IT with high sensitivity and specificity in 14 ventilated patients. However, as pointed out in an accompanying editorial [25], this method has the disadvantage of detecting only one type of patient-ventilator interaction. Younes *et al*. [26] monitored patient-ventilator interaction with a proprietary system that generates a signal mimicking respiratory muscle pressure output. The signal was derived from the equation of motion of the respiratory system using improvised values for resistance and elastance. This method could detect 80% of IT efforts when applied to airway signal tracings from 21 mechanically ventilated patients.

A problem common to the above methods is the distorting effect of background noise on the airway signals, a phenomenon that may affect their ability to distinguish small deflections indicative of wasted inspiratory effort. Moreover, these methods also may fail to identify conditions in which ventilatory support during inspiration is not sufficient to meet ventilatory requirements [27]. Although not totally immune to the effect of noise, the method presented here does not analyze airway signals for difficult to detect anomalies. Instead, it applies a Fourier transformation to several cycles of expiratory flow to produce a frequency spectrum. The shape of the resulting spectrum is determined by variations in breath-to-breath changes in T_Tot_. Regularity in T_Tot _is associated with sharply defined peaks that repeat at frequency multiples of respiratory rate. On the other hand, as T_Tot _becomes variable, the spectral pattern becomes less regular. These changes can be readily determined by visual inspection of the spectrum, or as shown in this study, by changes in the parameter H_1_/DC.

## Conclusions

The present study is the first report on the clinical application of spectral analysis of airway flow to identify the occurrence of asynchronous events in mechanically ventilated patients. Since the method is noninvasive, fully automatic and adaptable to existing ventilator monitoring systems, it may provide timely and actionable information on patient asynchrony both during invasive and non-invasive ventilation.

This is a preliminary, and by no means exhaustive, study on the use of spectral analysis of airway flow to characterize patient-ventilator asynchrony. It was not our purpose to ascertain the causes or treatment of asynchrony since this was an observational study in which all therapeutic and ventilator management decisions were determined by physicians who were not part of the research team. Further work remains to be done in validating the method, including clinical trials in which changes in H_1_/DC are compared to pressure changes obtained with esophageal balloon catheters. We also must learn its limitations. Further studies are needed to understand its utility in patients ventilated with assisted ventilatory modes.

Another limitation concerns the degree of intrinsic irregularity of respiratory pattern noted in alert individuals. Whereas a totally disorganized pattern appears to be indicative of severe asynchrony, a highly organized spectral pattern also may not be desirable, as it could indicate conditions that may also adversely affect the outcome [28], such as the excessive use of sedatives and neuromuscular blockade. Studies conducted in alert, mechanically ventilated patients who are synchronous with the ventilator are needed to establish the level of H_1_/DC separating physiologically appropriate T_Tot _variations ("good noise") from detrimental ventilator-patient asynchrony.

## Key messages

• Mechanically ventilated patients who fail to synchronize with the ventilator have worse outcomes.

• A reliable, non-invasive method of monitoring asynchrony on a real-time basis is not presently available.

• Spectral analysis of airway flow can detect asynchrony based on changes in the frequency spectrum.

• This noninvasive, fully automatic method to monitor asynchrony can be easily adapted to existing ventilator monitoring systems.

## Abbreviations

AI: Asynchrony Index (%); cpm: cycles per minute; DC: direct current component or zero frequency amplitude; FFT: Fast Fourier Transform; F_I_O_2: _inspired O_2 _concentration; GWU: George Washington University; H_1_: first harmonic peak amplitude; HR: heart rate; IT: ineffective triggering; MAP: mean arterial pressure; MIP: mean inspiratory pressure; Mode: mode of mechanical ventilation; n: number of patients; PeakP: peak inspiratory pressure; PC: pressure controlled; PEEP: positive end expiratory pressure; PS: pressure support; PRVC: pressure regulated volume control; ROC: receiver operating characteristic; RR: respiratory rate; SpO_2: _arterial O_2 _saturation by pulse oximetry; SAPS: Simplified Acute Physiology Score; T_Tot_: total breath cycle time; VC: volume controlled; V_T_: tidal volume.

## Competing interests

GG has submitted a patent application based on the method described in the manuscript. All other coauthors claim no competing interests.

## Authors' contributions

GG conceived of the study, participated in its design and coordination, performed the statistical analysis and was the main author of the manuscript. GJB participated in the design of the study, collection and analysis of data, and helped to draft the manuscript. HT and JA participated in the design of the study and helped to draft the manuscript. JA and VJ participated in the design of the study, collection of data and helped to draft the manuscript. LDLC and SG participated in the design of the study and collection of data. CE and BG participated in the collection and analysis of data and helped draft the manuscript. All authors have read and approved the final manuscript.
